# Baker’s Yeast Induces Apoptotic Effects and Histopathological Changes on Skin Tumors in Mice

**DOI:** 10.1080/2331205X.2018.1437673

**Published:** 2018-02-22

**Authors:** Amany Elwakkad, Mamdooh Ghoneum, Mamdouh El-sawi, Saadia Ibrahim Mohamed, Amina A. Gamal el Din, Deyu Pan, Ghada Mahmoud Elqattan

**Affiliations:** 1 Department of Medical Physiology, National Research Centre, Cairo, Egypt, Charles Drew University of Medicine and Science, Los Angeles, California, USA; 2 Department of Surgery, Charles Drew University of Medicine and Science, Los Angeles, California, USA; 3 Faculty of Science, University of Mansoura, Mansoura, Egypt; 4 Department of Pathology, National Research Centre, Cairo, Egypt; 5 Department of social and preventive medicine, Charles Drew University of Medicine and Science, Los Angeles, California, USA

**Keywords:** Skin cancer, Baker’s yeast, apoptosis

## Abstract

The current study investigates the apoptotic effect of Baker’s yeast (*S. cerevisiae*) on chemically-induced skin cancer in mice. Intra-tumoral treatment with yeast caused: increases in Ca^2+^ in skin homogenate, modulated the intrinsic/extrinsic pathways by downregulating Bcl-2 and FasL, upregulating Bax, and increased the expression of cytochrome-c and caspases 9, 8, and 3. Histopathological changes were detected, including mild dysplasia, atypia, tumor regression, and absence of basaloid cell proliferation. No toxic effects were detected, as examined by histopathological, biochemical, and body weight analysis. These results show that yeast exerts anti-skin cancer activity, suggesting its possible use for treatment of human skin cancer.

## Introduction

Skin cancer is the most common of all cancer types. In the United States, more than 87,000 cases of melanoma (the most serious form of skin cancer) are expected to be diagnosed in 2017 (1). In Australia, skin cancer is especially prevalent: by the age of 70, two in three Australians will have been diagnosed with skin cancer, and more than 434,000 Australians are treated for one or more non-melanoma skin cancers each year (2). Incidences of skin cancer are expected to increase worldwide, mainly due to excessive sunlight exposure, which contains harmful ultraviolet (UV) radiation, as well as changes in dietary habits and lifestyle. Treatment of skin cancer can involve surgery, chemotherapy, and radiation therapy, and combination therapy is always preferred over a single treatment modality.

Though several treatments have been discovered for skin cancer, including chemotherapy (3,4) and interferon alpha (IFN-α) and its pegylated forms, these are known to have severe side effects (5). While new cancer immunotherapy (immune checkpoint inhibitors) represents a possible breakthrough for treatment (6–8), most cancer patients, including melanoma patients, developed immune-related adverse events (9,10). Unfortunately, current treatments are not enough to effectively and safely combat the disease. Therefore, the need for new agents that exert anti-skin cancer activity with minimal side effects has become an emerging area of research.

Another method that has been used to treat skin cancer is the injection of microbes, including *Corynebacterium parvum* (*C. parvum*) and Bacillus calmette Guérin (BCG) (11–13). However, the clinical use of these BRMs has been severely limited because of their *in vivo* cytotoxicity (14–17). In the current study, we examined the effect of a safe microbe that is a known food supplement, baker’s yeast, *Saccharomyces cerevisiae* (*S. cerevisiae*), for possible use as an anticancer agent against skin cancer in mice. *S. cerevisiae* is an essential component for the production of fermented foods like bread and beer. Our earlier studies showed that heat-killed baker’s yeast exerts anticancer activity against human breast cancer cells. This is based on the observation that cancer cells phagocytize yeast and that yeast then subsequently triggers apoptosis in cancer cells *in vitro* and *in vivo* (18,19). This study aims to assess baker’s yeast as a novel natural product that can induce an apoptotic effect, associated with histopathological changes, on skin cancer in animal bearing tumor. Results show anticancer effects of yeast treatment as a safe, non-toxic agent and may suggest its possible use as treatment against skin cancer in humans.

## Materials and Methods

### Animals

One hundred male Swiss albino mice aged 6–7 weeks (20±2 g body weight) were used in this study. The animals were obtained from the animal house at National Research Center, Giza, Egypt, and acclimatized for one week prior to the commencement of the study. Mice were housed in plastic cages and allowed to acclimate in standard conditions (under 12 light\dark cycle). The animals were given free access to distilled water and commercialized food throughout the experiment. The environmental conditions were standardized with respect to temperature, humidity, and light. This study was approved by the Animal Ethical Committee at National Research Centre, Cairo, Egypt.

### Preparation of S. cerevisiae

Baker’s and brewer’s yeast, *S. cerevisiae*, was purchased from ACH Food Companies, INK. Memphis, TN, USA. The yeast was used in suspensions that were washed once with Hanks balanced salt solution (HBSS). The suspensions were heat-killed *via* incubation for 1hr at 90°C and subsequently washed twice with HBSS. Quantification was carried out using a hemocytometer. Yeast cell suspensions were adjusted to 1×10^7^, 10^8^, and 10^9^ cells/ml and mice received IT injection of 100μL yeast.

### Tumor Induction

Mice were shaved on the dorsal skin using an electrical clipper and painted once with 7,12dimethylbenz[α] anthracene (DMBA) on one side (120.0 nmol DMBA in 0.2 ml acetone). One week later, mice were painted with 12-O-tetradecanoylphorbol 13-aceteate (TPA) twice weekly for 14 weeks (4.0 nmol TPA in 0.2 ml acetone) [20]. DMBA and TPA were purchased from Abcam. Inc. Cambridge, MA, USA.

When the tumor volume reached ~100 mm^3^, the mice bearing tumor alone without yeast treatment were sacrificed, along with the normal control mice without tumor. The rest of mice were injected intra-tumorally (IT) for 16 weeks with 100 μl yeast (2 times/week) at three concentrations: 10^7^, 10^8^, and 10^9^ cells/ml of yeast respectively, and then sacrificed. Each skin tumor was injected with 100μl of yeast. Thus, an animal bearing 2 skin tumors received 200μl of yeast treatment, 100μl into each tumor site.

### Experimental Design

This study comprised 100 mice, divided into 5 groups (20 mice/group) as follows: Group 1 served as normal control mice without tumors, Group 2 were mice bearing tumors, and Groups 3–5 were mice bearing tumors receiving yeast through IT injection 100 μl (2 times/week) at concentrations of 10^7^, 10^8^, and 10^9^ cells/ml, respectively. The mice in Group 2 were sacrificed when the tumor volume reached ~100 mm^3^; roughly two weeks following tumor induction. From our previous studies, this size of tumor has been shown to be an optimal tumor volume suitable for treatment with yeast. Delaying yeast treatment results in devastating rapid growth of the tumor and subsequently the animal dies within a few days. The rest of the mice bearing tumors in Groups 3–5 were sacrificed at 16 weeks post treatment with yeast. The tumor volume was measured horizontally and vertically by using digital caliper. Volume of the tumor (V) was determined by Carlsson V= (ab^2^)/2 where ‘a’ and ‘b’ are the longest and shortest diameters of the tumor, respectively. For these 5 groups, the following investigations of biochemical analysis for the apoptotic pathway were carried out: calcium ions, FasL, Bcl2, Bax, cytochrome-c, and quantification of caspases 8, 9 and 3. In addition, toxicity studies involving histopathological, biochemical analysis, and changes in body weight were completed.

### Preparation of Tumor/Skin Homogenates

Tumor homogenates were prepared as follows: mice bearing tumor, with and without yeast treatment, were sacrificed, samples of tumor tissue were cut, and weight of the tissues were recorded. Tumor tissues were then rinsed with ice-cold PBS (0.01M, pH=7.4), weighed, minced, and then homogenized in PBS with a glass homogenizer on ice (9mL PBS per gram tissue). The homogenates were centrifuged at 3000rpm for 10min and supernatant was removed and frozen at −70°C. Similarly, normal control mice without tumor were shaved and samples of skin (~100 mm^3^) were treated as above.

### Biochemical Analysis for the Apoptotic Pathway

The homogenates of tumor tissues under different treatment conditions and skin homogenates of normal control mice were analyzed for the levels of different parameters of apoptotic pathway: calcium ions, cytochrome-c, FasL, Bcl-2, Bax, and Caspases 8, 9, and 3, using enzyme-linked immunosorbent assay (ELISA) (Sun Red Biotechnology Company, Shanghai).

#### Calcium Ion Concentration.

Calcium ion levels were analyzed with a Calcium Ionized Detection ELISA Kit (Kono Biotech Co., Ltd.) according to the manufacturer’s instructions. Monoclonal antibodies were incubated with skin homogenate, followed by the addition of Calcium binding protein antibodies labeled with biotin and combined with Streptavidin-HRP to form immune complex. Incubation was then carried out and antibodies were washed again to remove the uncombined enzyme before Chromogen Solution A and B were added. The chroma of color and the concent Humanion of the different homogenate of Calcium Binding Protein B of sample were positively correlated. Calcium concentration in the supernatant materials was estimated according to the ELISA Kit datasheet.

#### Cytochrome-c, FasL, Bcl2, Bax, and Caspases.

Homogenates of tumor and healthy skin tissues from mice were analyzed using ELISA. The levels of cytochrome-c, FasL, Bcl2, Bax, and caspases 3, 8, and 9 activities were measured according to the ELISA kit instructions (Sun Red Biotechnology Company, Shanghai, People’s Republic of China).

#### Histopathological Analysis of Skin Cancer

At the end of the experiment, animals were sacrificed. The skin samples from negative control and animal bearing tumors, with and without yeast treatment, were excised and fixed in 10% buffered formalin, processed through ascending grades of alcohol, cleared in xylene and prepared into paraffin blocks. Serial sections 4 microns thick were prepared and stained with hematoxylin and eosin (H&E) for routine histopathological study. The sections were examined for histopathological changes using an Olympus CX41 microscope. Slide tissue microphotography was done using CCD digital camera Olympus DP-12 attached to the Olympus CX41 research microscope. Digital photomicrographic sections were taken at various magnifications.

#### Toxicity Studies

##### A-Histopathological analysis

Normal control mice and mice bearing tumor were treated IT with yeast (100 μl) at different concentrations (10^7^, 10^8^, 10^9^ cells/ml) for 16 weeks. Mice were then killed and different organs (liver, kidney, and testis) were excised and sections were prepared and stained with H&E for histopathological analysis.

##### B-Biochemical analysis

Biochemical analysis in healthy control mice with and without yeast treatment was carried out. Biochemical analysis of liver function was analyzed by serum aspartate aminotransferase (AST), serum alanine aminotransferase (ALT), and alkaline phosphatase (ALP), while kidney function was measured by creatinine levels. Other parameters, including blood urea and lipid peroxidase were examined. All the above mentioned assays were conducted using colorimetric determination by the Biodiagnostic Kit, Egypt. We used previously described methods to determine AST and ALT levels (21), Lipid Peroxide quantification (22), creatinine levels in serum (23), and Alkaline Phosphatase levels (24). The blood urea was determined according to the Urease-Berthelot method (25).

##### Statistical Analysis

All statistics were performed using SPSS 14 for Windows package (SPSS Inc., Chicago, IL, USA). The differences between means were performed using student “t” test and the comparison of the study groups was done by using analysis of variance (ANOVA), followed by Dunnett post hoc comparisons. This was done to compare changes between groups of the study and the data obtained in the present work were represented in tables as average (Mean) ± Standard Error. In all tests *P* value was considered significant when less than or equal 0.05 and highly significant when less than or equal 0.01.

## Results

### 1. Biochemical analysis for the apoptotic pathway.

#### 1.1. Calcium ions.

**Figure 1:**
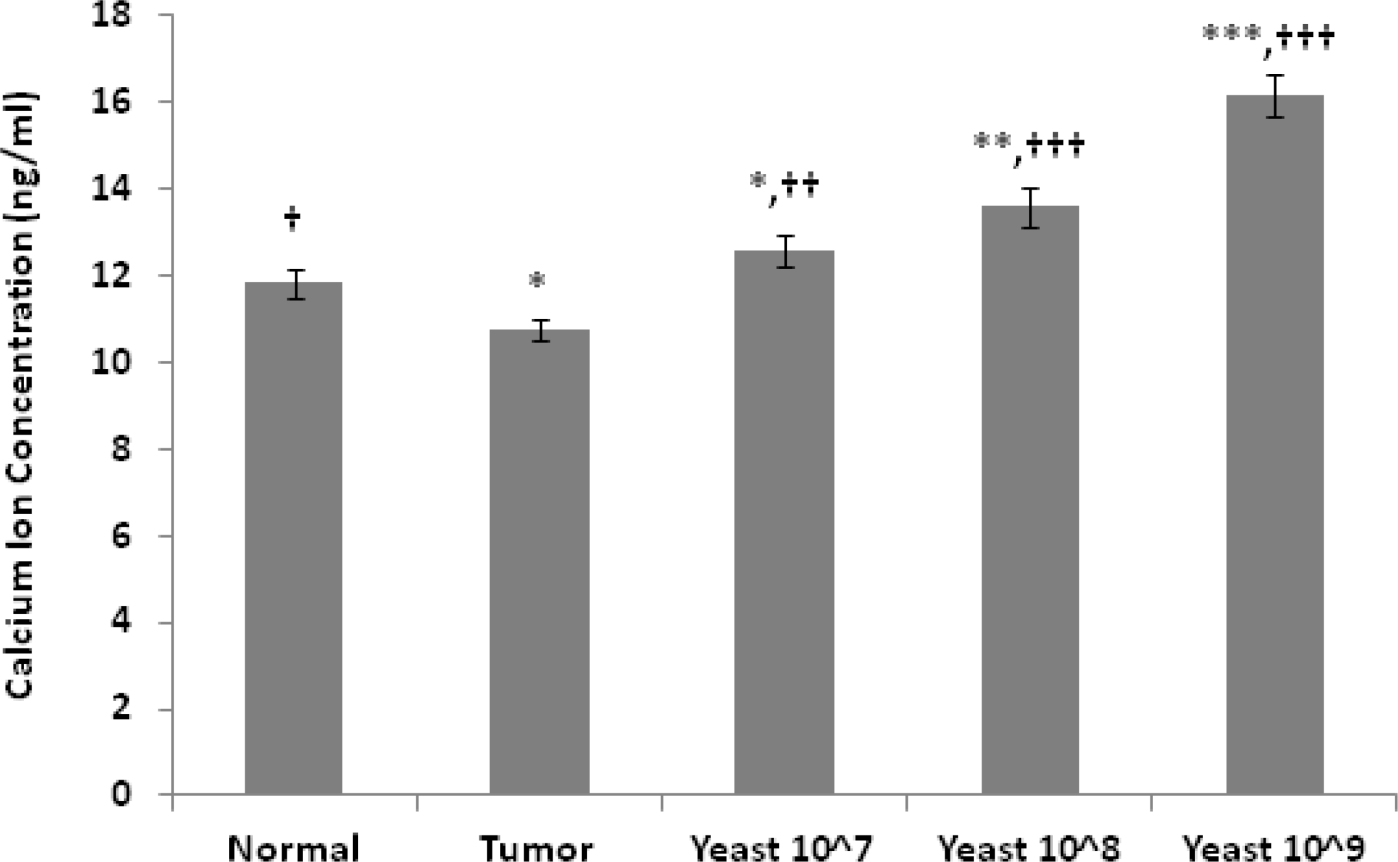
Changes in calcium ion concentrations in mice under different treatment conditions: normal control mice, untreated tumor bearing mice, and tumor bearing mice treated with yeast at three concentrations of 10^7^, 10^8^ and 10^9^ cells/ml. Data represent Mean±S.E. from 20 mice per group. (Significance relative to normal control mice: *p≤0.05, **p≤0.01, ***p≤0.001; Significance relative to untreated tumor bearing mice: †*p*≤0.05, ††*p*≤0.01, †††*p*≤0.001).

Data in Figure 1 shows a significant decrease in the concentration of ionized calcium (Ca^2+^) in the skin homogenate of tumor-bearing mice as compared to normal control mice (*p≤*0.05). However, treatment with yeast resulted in an increase in the concentration of ionized calcium in the skin homogenate of mice that received 100 μl yeast at concentrations 10^7^ (*p≤*0.01), 10^8^ (*p≤*0.001), and 10^9^ (*p≤*0.001) in comparison to tumorbearing mice.

#### 1.2. Analysis of Apoptotic Regulators.

The expression of pro-apoptotic proteins FasL and Bax and anti-apoptotic protein Bcl-2 were examined in mice under different treatment conditions: normal control skin, tumor skin alone, and yeast-treated (100 μl) tumor skin at concentrations 10^7^, 10^8^, and 10^9^ cells/ml.

##### FasL.

**Figure 2:**
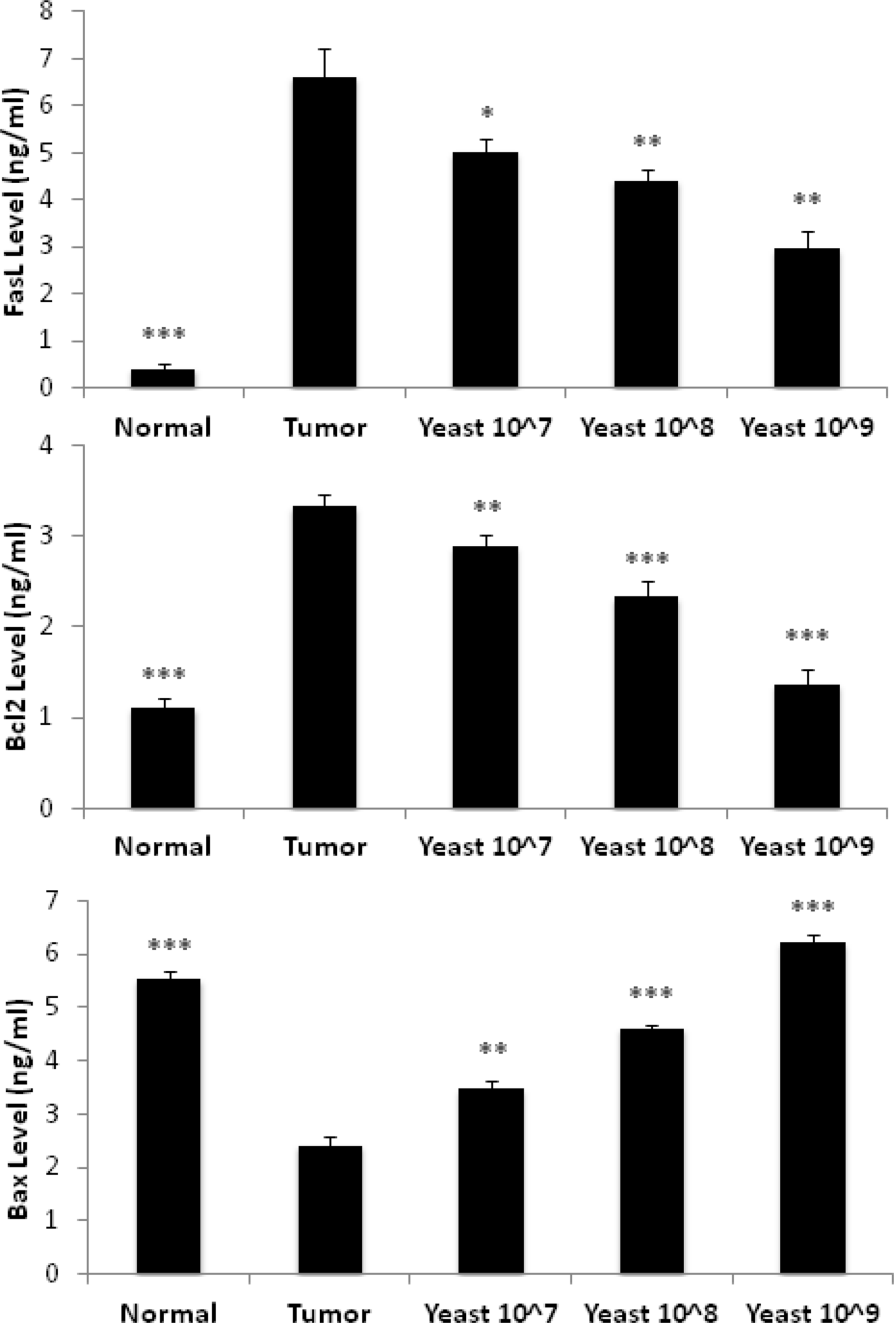
Effect of IT injection with yeast on FasL, Bcl2, and Bax levels in normal control mice, untreated tumor bearing mice, and tumor bearing mice treated with yeast at three concentrations of 10^7^, 10^8^ and 10^9^ cells/ml. Data represent Mean±S.E. from 20 mice per group. (Significance relative to untreated tumor bearing mice: *p≤0.05, **p≤0.01 and ***p≤0.001).

Figure 2 shows a significant increase in the level of FasL in tumor homogenate in mice bearing tumor alone as compared with normal control skin homogenate (*P*≤0.001). In contrast, the expression of FasL significantly decreased post treatment with yeast in a dosedependent manner: 10^7^ (*p≤*0.05), 10^8^ (*p≤*0.01), and 10^9^ (*p≤*0.01) in comparison to tumorbearing mice alone.

##### Bcl2.

The expression Bcl2 was significantly increased in tumor homogenate in comparison with normal control tissue (*p*≤0.001). However, treatment with yeast caused a decrease in Bcl2 expression in a dose-dependent fashion when compared with animal bearing skin cancer alone. A decrease in Bcl2 expression was detected post treatment with 100 μl yeast concentration of 10^7^ (*P***≤**0.01), and the effect became highly significant at concentrations of 10^8^ and 10^9^ cells/ml in comparison to tumor skin homogenate (*P***≤**0.001) (Figure 2).

##### Bax.

Data in Figure 2 shows that the Bax level was significantly decreased in tumor homogenate in animal bearing tumor in comparison with normal control tissue (*p*≤0.001), while treatment with yeast caused an increase in Bax level in a dose-dependent fashion when compared with animal bearing skin cancer.

#### 1.3. Cytochrome-c.

**Figure 3:**
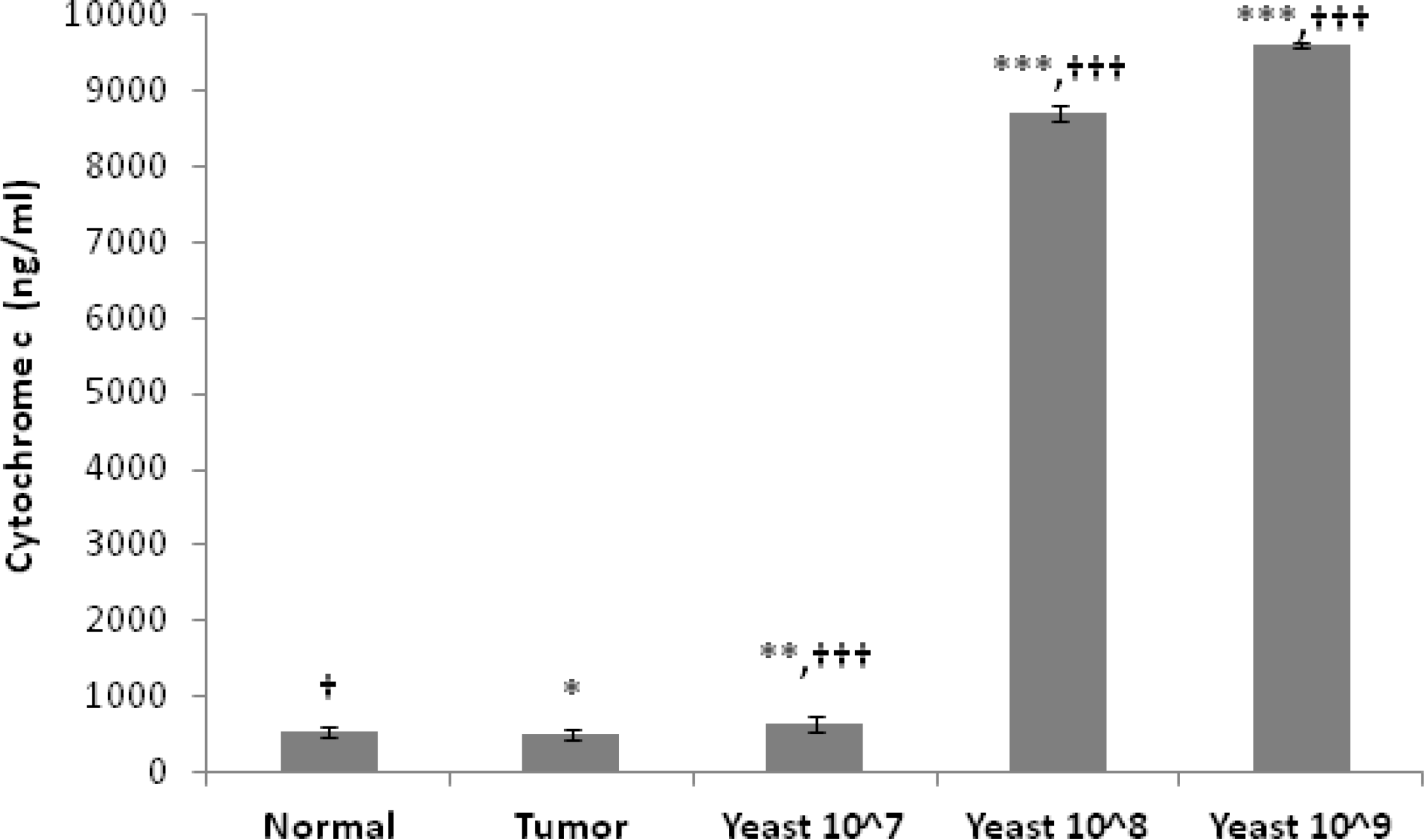
Effect of IT injection with yeast on the levels of cytochrome-c in normal control mice, untreated tumor bearing mice, and yeast-treated tumor bearing mice. Data represent Mean±S.E. from 20 mice per group. (Significance relative to normal control mice: *p≤0.05, **p≤0.01, ***p≤0.001; Significance relative to untreated tumor bearing mice: †*p*≤0.05, †††*p*≤0.001).

Results depicted in Figure 3 show a slight difference in the levels of cytochrome-c (pg/ml) between homogenates of normal control skin and tumor (*p≤*0.05). Treatment with 100 μl yeast at low concentration (10^7^cells/ml) resulted in a slight increase in the level of cytochrome-c. However, higher concentrations of 10^8^ and 10^9^ cells/ml resulted in a dramatic increase in cytochrome-c by over 15 fold (*p*≤0.001).

#### 1.4. Quantification of Caspases 9, 8 and 3.

**Figure 4:**
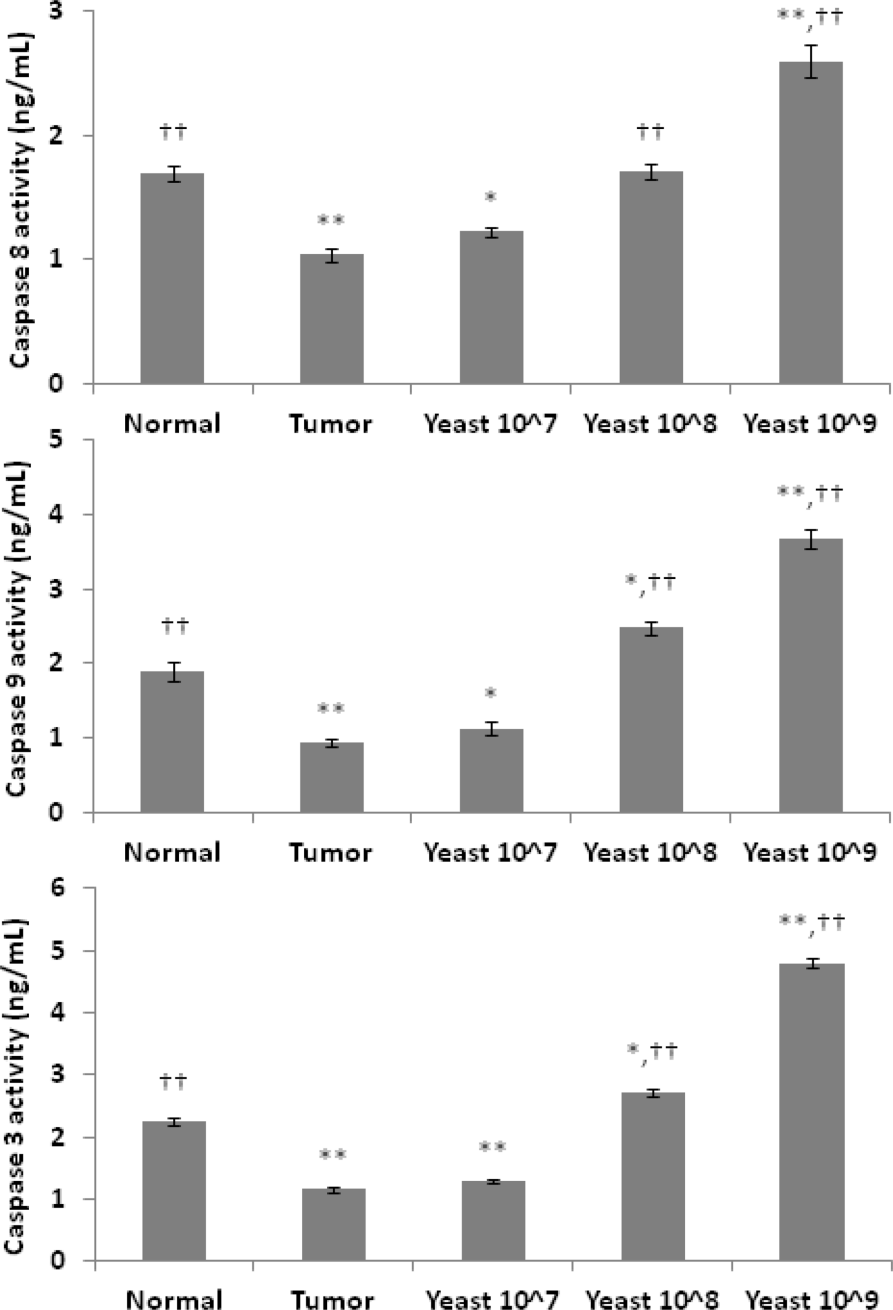
Levels of caspases 8, 9, and 3 in skin cancer cell post treatment with yeast. Mice bearing tumor received IT injection with yeast at concentrations of 10^7^, 10^8^, and 10^9^ cells/ml, and the level of caspases 8, 9, and 3 were examined by ELISA. Data represent Mean±S.E. from 20 mice per group. (Significance relative to normal control mice: *p≤0.05 and **p≤0.01; Significance relative to untreated tumor bearing mice: ††*p*≤0.01).

The level of caspases 9, 8, and 3 were examined in mice bearing tumor and after treatment with yeast. Mice bearing tumor demonstrated a decrease in the level of caspases 9, 8, and 3 as compared with normal control group. On the other hand, treatment with yeast resulted in an increase in the expression of caspases 9, 8, and 3 in a dose-dependent fashion (Figure 4).

### 2. Histopathological analysis.

Histopathological changes were examined in tumor-bearing mice with and without yeast treatment.

#### 2.1. Skin of normal healthy mice and animal bearing tumor.

**Figure 5 A-F:**
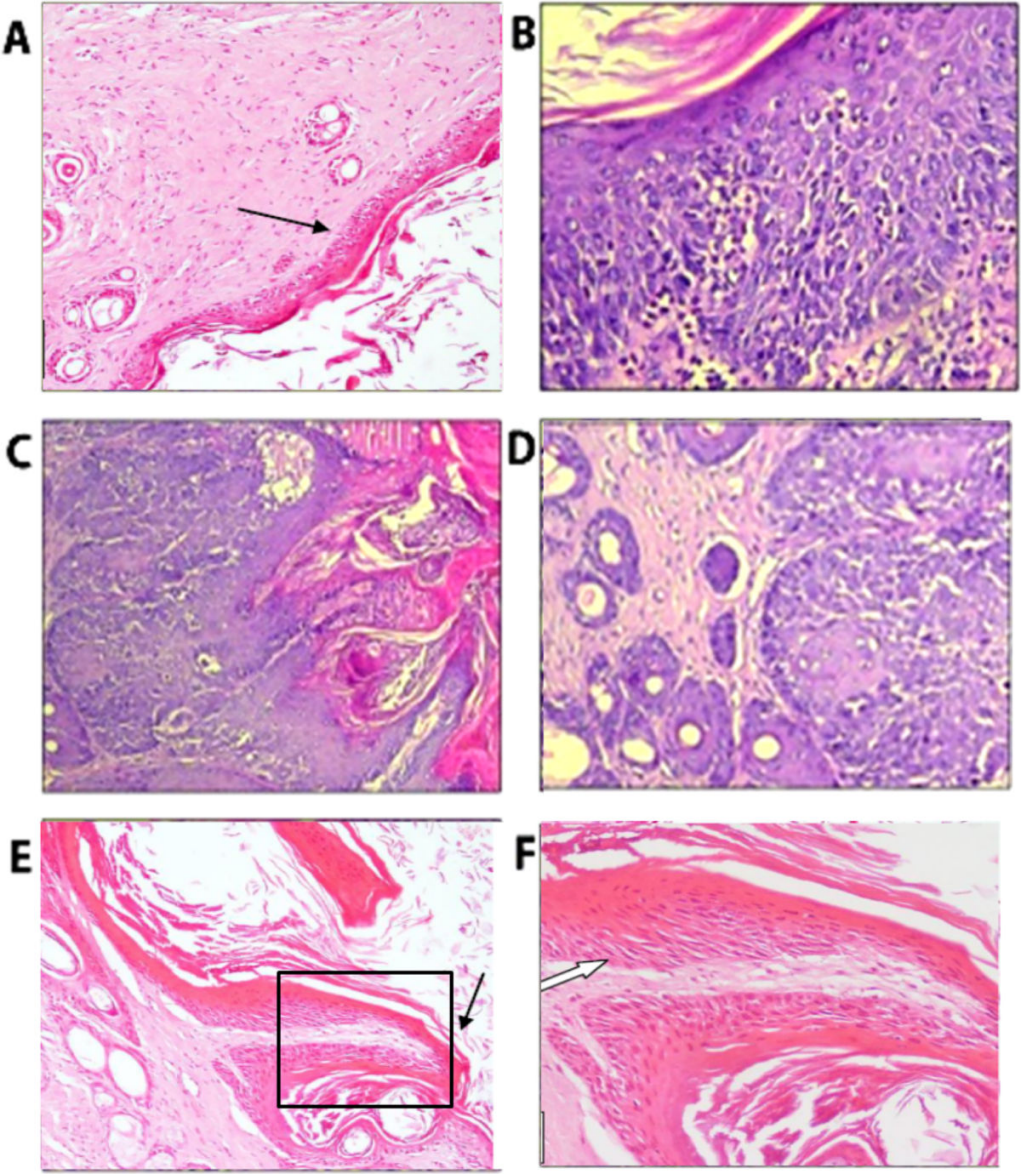
Histopathological examination of H&E-stained skin tissues from normal healthy mice and tumor skin alone. (A) shows skin tissue of normal healthy mice covering ordinary stratified squamous epithelium (black arrow). (x400); (B) Tumor tissue showing epidermal acanthosis and moderate dysplasia with underlying inflammation (x200); (C) Tumor skin showing epidermal papillae with hyperkeratosis, bulbous ends and prominent dysplasia (x100); (D) The lower bulbous end of the epidermal acanthotic papillae with starting anaplastic changes (x200); (E). Skin tumor tissue showing surface papillary projection (black arrow) (x200). (F) Magnified view of black square on E showing surface papillary projection with mild acanthosis, hyperkeratosis and focal mild dysplasia within lining epithelium (white arrow) (x400).

Figure 5A shows skin from normal healthy mice while Figure 5B–5F shows skin from tumor-bearing mice. In figure 5A, healthy mouse skin shows ordinary benign stratified squamous epithelium with the underling dermal connective tissues showing adnexal structures. The skin of tumor-bearing mice in Figure 5B shows marked epidermal-epithelial proliferation and hyperplasia. Moderate dysplasia with dense chronic inflammatory infiltrate can be seen underneath the covering of proliferating epidermis at the epidermal–dermal junction. Figure 5C shows epidermal papilla hyper-keratosis, elongated rete ridges, bulbous ends, and prominent dysplasia. In addition, the focus of a crater-like ulcer was seen filled with keratin with an adjacent overhanging epidermal edge (Figure 5D). This appearance was consistent with starting early malignant changes in the form of low grade, well-differentiated squamous cell carcinoma. Surface capillary projection of tumor tissue can be seen in Figures 5E and 5F with mild acanthosis, hyperkeratosis, and focal mild dysplasia within lining epithelium.

#### 2.2. Tumor skin of mice treated with yeast.

**Figure 6 A-E:**
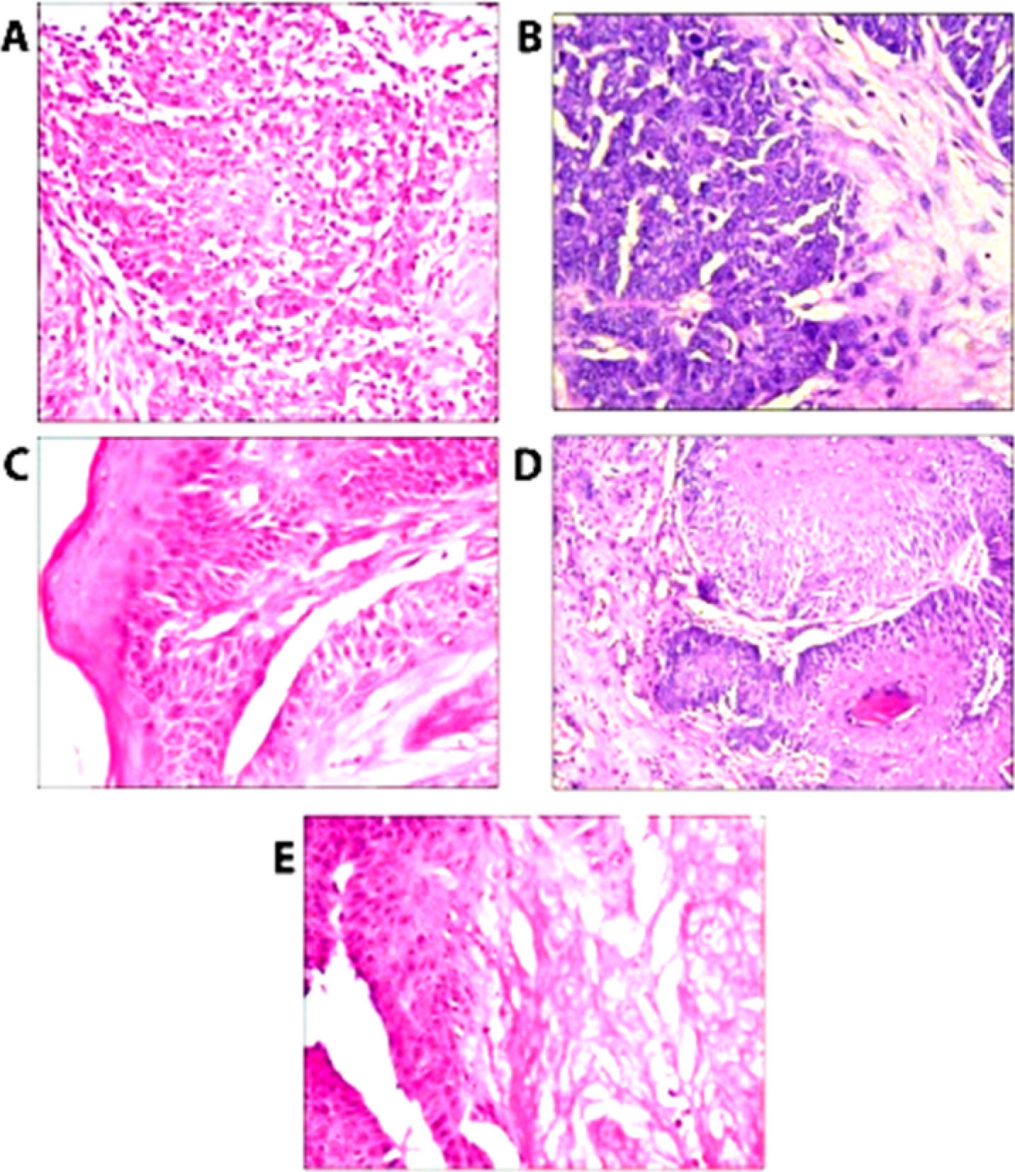
Histopathological examination of H&E-stained tumor tissue from yeast-treated mice. (A&B) Skin of mice showed dense inflammatory aggregates infiltrating lobules of mildly proliferating basaloid cells with diminished atypia (x200). (C&D) showing a decrease of epidermal cell lining and mild dysplasia was noticed. Mitosis, polymorphism and atypia were diminished (x200). (E) Showing marked diminution of the thickness of epidermal layers without atypia (x200).

Figure 6 illustrates histopathology of the tumor skin treated with 100 μL of yeast at concentrations of 10^7^, 10^8^, and 10^9^ yeast/ml. Treatment with a concentration of 10^7^ yeast/ml resulted in dense inflammatory cell aggregates infiltrating lobules of mildly proliferating basaloid cells with atypia (A and B). After treatment with 10^8^ yeast/ml, there was a decrease in the thickness of the epidermal cell lining being formed of differentiated keratinocytes, and mild dysplasia was noticed. Mitosis, polymorphism, and atypia were diminished. Tongues of proliferating basaloid cells invading the dermis were minimal, and dermal inflammation was also diminished (C and D). Treatment with yeast at higher concentration of 10^9^ yeast/ml caused tumor regression (E). There was also a decrease in the thickness of the epidermal cell layers. Lining consisted of well-differentiated keratinocytes and foci of glassy keratinocytes were observed. Diminished keratin was also seen. We noticed the absence of basaloid cell proliferation, tongues, lobules, and cords of basaloid cells invading the dermis in this group. Atypia was markedly regressed with absence of dysplasia, mitosis and polymorphism.

### 3. Toxicity

Toxicity studies were carried out in order to determine if yeast treatment is safe to use. The following parameters were examined: A) animal behavior, B) animal survival, C) histopathological analysis of internal organs, and D) biochemical analysis.

#### A. Monitoring animal behavior

Animals were monitored to observe potential toxic side effects of yeast treatment. Daily examinations showed that injections of heat-killed *S. cerevisiae* gave no adverse side effects as indicated by the fact that life activity patterns, including feeding/drinking of yeast-treated mice, were recorded for the entire treatment period and showed to be normal as compared with normal control.

#### B. Monitoring animal survival

**Figure 7A-D:**
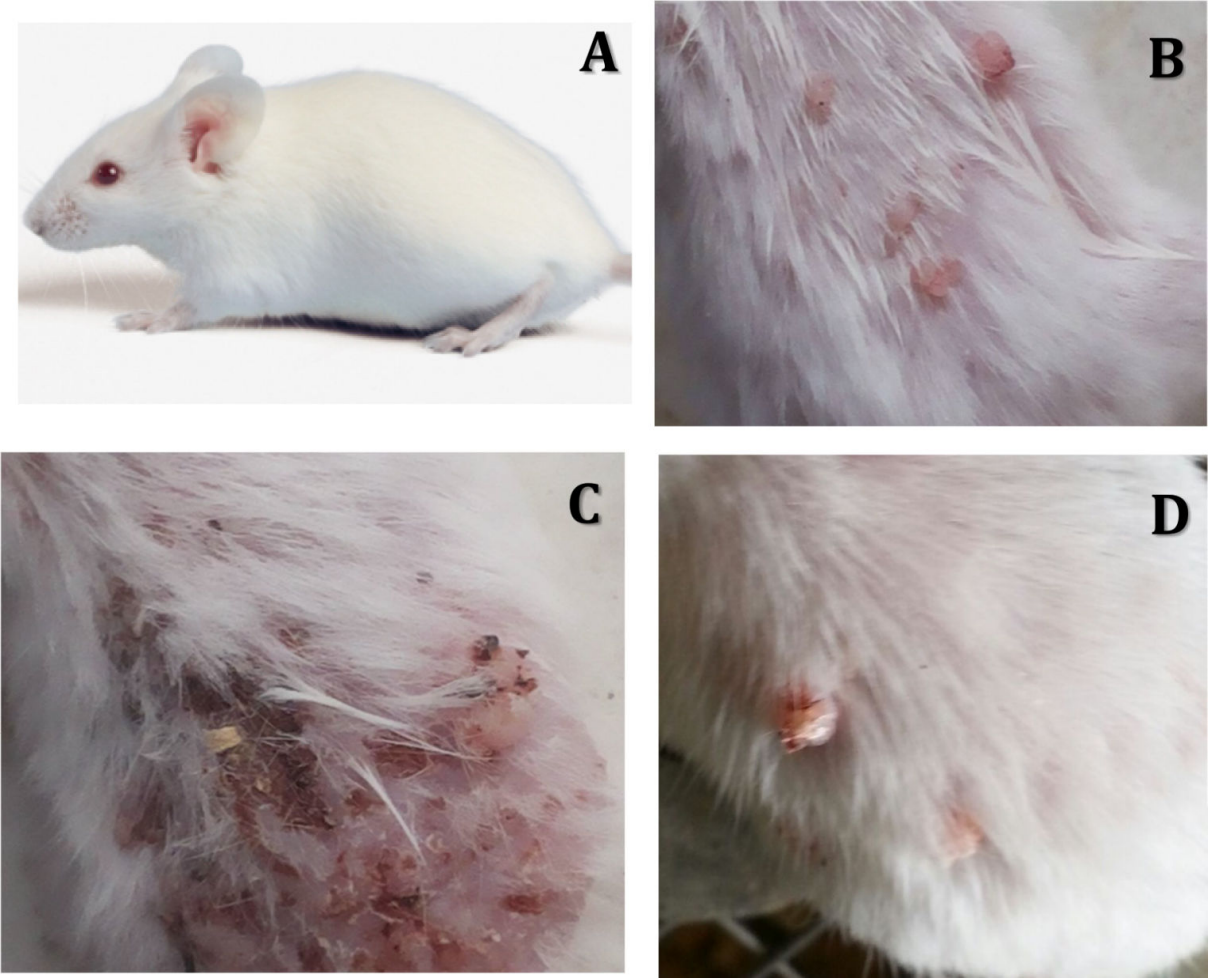
Effect of yeast treatment on skin cancer. (A) Healthy mice. (B) Mice treated with DMBA and TPA for 14 weeks. Visible skin tumors formed. (C) Mice with tumor tissue left yeast-untreated for 2–3 weeks worsened, prompting mice to be sacrificed. (D) Tumor tissues receiving yeast injections for 16 weeks. Notice tumor tissue has receded significantly.

Results of this study showed that all animals receiving yeast treatment survived for the duration of the treatment period of 16 weeks. On the other hand, mice bearing skin tumor without yeast survived for only 2–3 weeks. Figure 7A-D shows mice under different treatment conditions. (A) shows healthy mice, (B) shows mice bearing tumor post treatment with DMBA and TPA for 14 weeks, (C) shows mice bearing tumor left untreated for 2–3 weeks with significant expansion and worsening of tumor tissue, (D) shows mice bearing tumor receiving yeast treatments (100 μl at a concentration of 10^9^ yeast) for 16 weeks with significantly fewer traces of cancer seen.

**Figure 8A-D:**
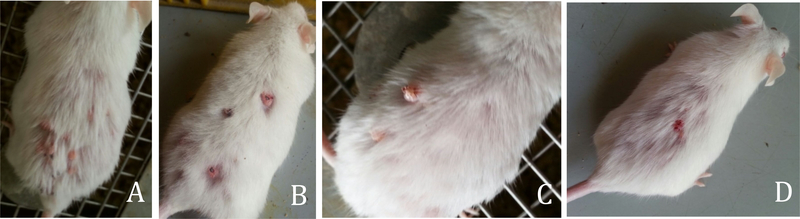
Dose-response relationship between yeast treatment and tumor growth. (A & B) Mice treated with low dose (100 μl at a concentration of 10^7^ yeast) show multiple nodual. (C & D) Mice treated with higher dose (100 μl at a concentration of 10^9^ yeast) show significantly fewer noduals.

Figure 8A-D shows a dose-dependent relationship between yeast treatment and tumor growth. Mice treated with a low dose of yeast (100 μl at a concentration of 10^7^ yeast) showed multiple noduals (A & B). Mice treated with a higher dose of yeast (100 μl at a concentration of 10^9^ yeast) resulted in a significant reduction in tumor noduals (C & D).

#### C. Histopathological Analysis

**Figure 9:**
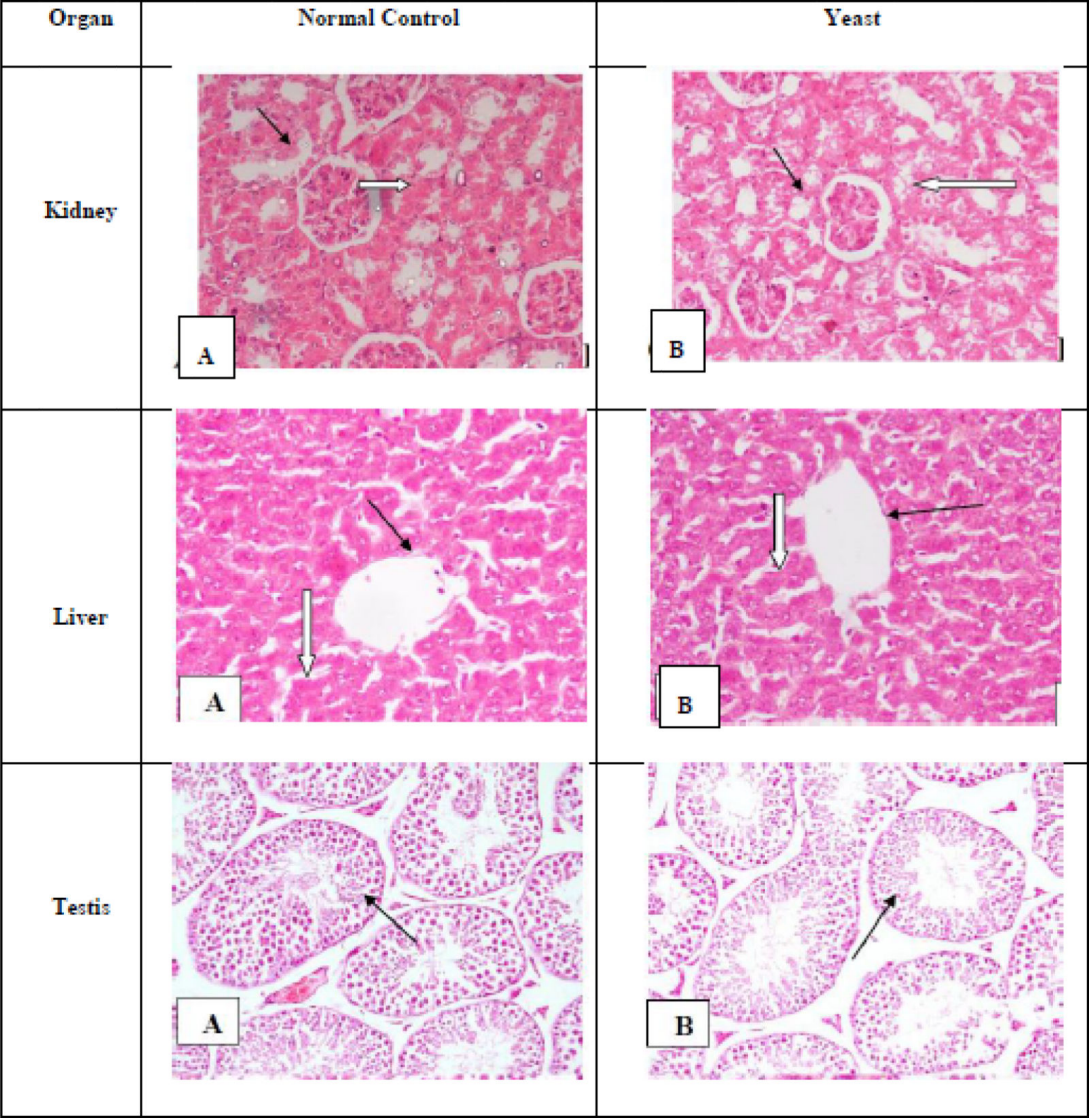
Histopathological examination of H&E-stained different organs from normal control mice and mice bearing tumor treated with yeast (100 μl at a concentration of 10^9^ yeast) (x200 magnification). Kidney: (A) Kidney tissue of control mouse showing average cellular, ordinary glomeruli (black arrows) and surrounding renal tubules lined by low cuboidal epithelium (white arrows). (B) Kidney tissue of yeast-treated mice showing average cellular glomeruli (black arrow). Scattered renal tubules showed mild vacuolar degeneration within lining epithelium (white arrows). Liver: (A) Liver tissue of control mouse showing preserved architecture with central vein (black arrow) and hepatocytes radiating from it, polyhedral, disposed in cords (white arrow). (B) Liver tissue of yeast-treated mice showing preserved architecture with ordinary hepatocytes (white arrow) radiating from central vein (black arrow). Testis: (A) Testicular tissues of control mouse showing ordinary seminiferous tubules (black arrow) of average caliber, basement membrane with average thickness and lined by cells at all stages of spermatogenic series. (B) Testicular tissue of yeast-treated mouse showing ordinary seminiferous tubules (black arrow) approximating control.

At the end of the experiment, normal control mice and tumor-bearing mice treated with yeast (100 μl at a concentration of 10^9^ yeast) were sacrificed. Kidney, liver, and testis were dissected and extracted from sacrificed animals. The H&E sections were examined under light microscope. Figure 9 shows that histology sections from yeast-treated mice had normal appearances comparable to those of healthy control mice without treatment.

#### D. Biochemical Analysis

Results depicted in Table 1 revealed that mice-bearing tumor showed significantly higher levels of AST, ALT, ALP, urea, and creatinine as compared with normal mice. However, treatment with yeast reversed the levels of these parameters to the values of normal mice. Furthermore data in Table 1 showed significant differences among mouse groups receiving different yeast doses, for example: (1) the levels of AST U/l: Yeast 10^9 vs. Yeast 10^7, *p≤0.05*, (2) the levels of ALT U/l: Yeast 10^9 vs. Yeast 10^8, *p≤0.01*, and (3) the levels of ALT U/l: Yeast 10^9 vs. Yeast 10^7, *p≤0.01*.

Results in Table 2 show that animals bearing tumor had a 15-fold increase in lipid peroxidase levels, however treatment with yeast decreased the levels of lipid peroxidase in a dose-dependent manner and reached the limits of the normal control values at the high dose of yeast (100 μL yeast at a concentration of 10^9^ cells/ml).

#### E. Body Weight (BW)

**Figure 10:**
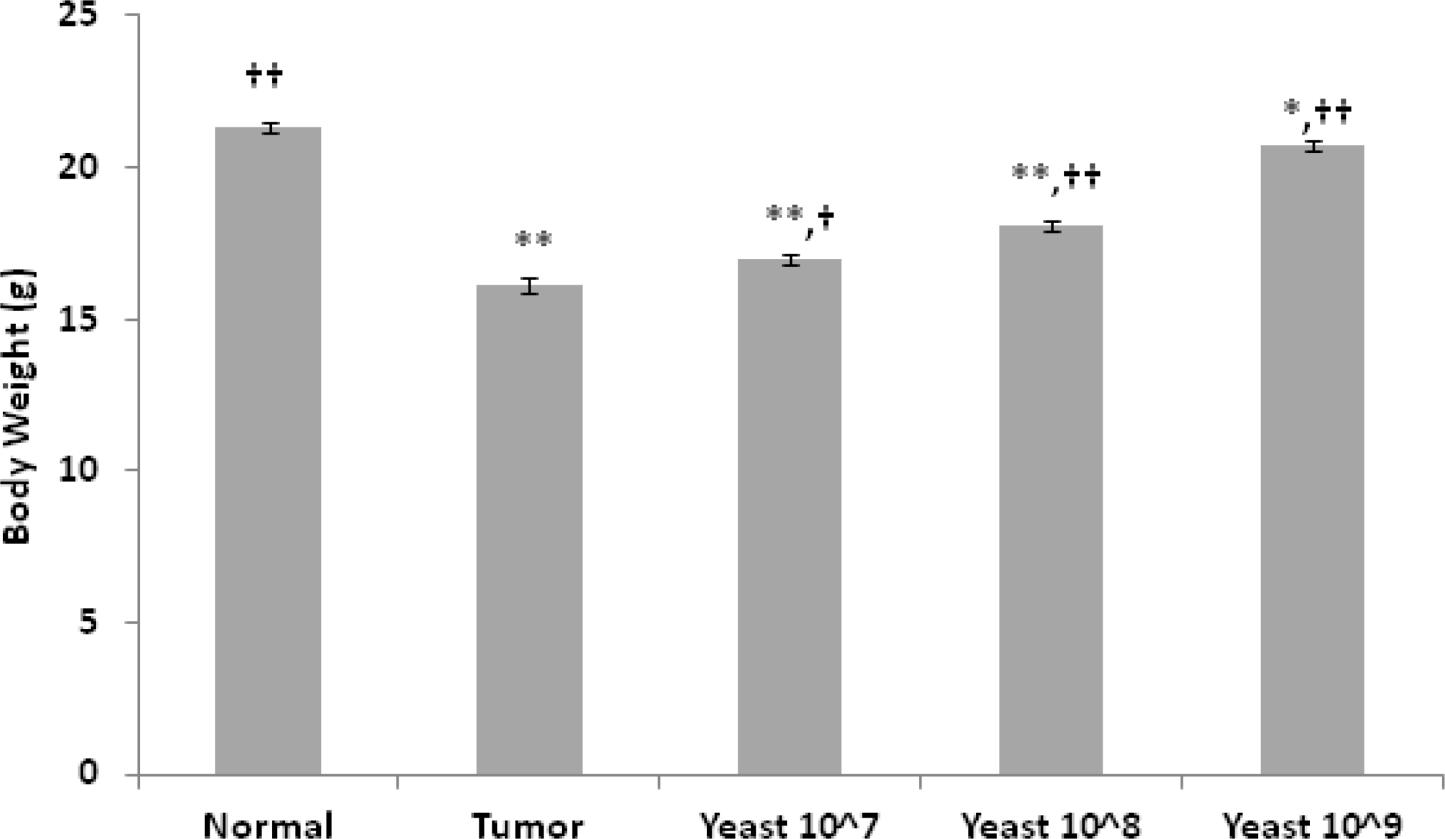
Changes in mice body weight (g) (mean ± S.E.) under different treatment conditions: normal control mice, untreated tumor bearing mice, and tumor bearing mice treated with yeast at three concentrations 10^7^, 10^8^, and 10^9^ cells/ml. Data represent Mean±S.E. from 20 mice per group. (Significance relative to normal control mice: *p≤0.05, **p≤0.01; Significance relative to untreated tumor bearing mice: †p≤0.05, ††p≤0.01).

Changes in BW in mice under different treatment conditions were examined. As shown in Figure 10, animals bearing skin tumor demonstrated a significant decrease in body weight as compared to normal control mice (*p*≤0.001). On the other hand, IT injection of yeast (100 μl) resulted in a significant gain in BW. The effect of yeast treatment was dose dependent. The effect was less remarkable with lower concentrations (10^7^ cells/ml) and maximized at a concentration of 10^9^ cells/ml, bringing the body weight close to the normal control mice.

## Discussion

The ability of Baker’s yeast (*S. cerevisiae*) to trigger cancer cell apoptosis is one of the most remarkable features of antitumor activity shown in our lab. We have previouly shown the apoptotic effect of heat-killed Baker’s yeast against several human cancer cell lines of the breast, tongue, and colon *in vitro* (18,26–28), in nude mice bearing human breast cancer, and Swiss albino mice bearing Ehrlich Ascites Carcinoma (EAC) cells (19,29,30), and without inducing a significant effect in normal cells (21). Results of this study show the ability of yeast to induce apoptosis in skin cancer in mice bearing tumors. Yeast-injection IT (100 μl) for 16 weeks at concentrations of 10^7^, 10^8^, and 10^9^ cells/ml caused apoptosis of cancer cells in a dose-dependent manner via modulation of the level of the Bcl-2 family, downregulating FasL levels, and activation of caspases.

Microbes, including Corynebacterium parvum (C. parvum) and Bacillus calmette Guérin (BCG), have been used to treat skin cancer (11–13). C. parvum and BCG act as immune modulators as manifested by activation of murine splenic macrophages via type I interferon production (31). It has been suggested that T cells play a key role in the activation of macrophages (32). These microbes also trigger apoptosis in cancer cells via TLRs (33).

Calcium ions (Ca^2+^) play a vital role in the induction of apoptosis (34,35). The endoplasmic reticulum (ER) is a major storage site for calcium. During phagocytosis, intracellular calcium rises after being released from the ER (36–39). In the current study we examined the concentration of Ca^2+^ in tumor homogenates with and without yeast treatment. Results demonstrated a significant decrease in the concentration of Ca^2+^ in animal bearing skin cancer as compared to skin from normal control mice. However, treatment with yeast IT resulted in a significant increase in the concentration of Ca^2+^, relative to animals with tumor alone, in a dose-dependent manner. This data is in accordance with our earlier *in vitro* studies, which showed that yeast-induced apoptosis against human metastatic breast cancer cells, MDA-MB-231, was Ca^2+^ dependent (40). This was indicated by the elevation of Ca^2+^ post culture of cancer cells with yeast, and by a significant suppression of apoptosis of cancer cells upon addition of 2-aminoethoxydiphenyl borate (2APB), a pharmacological inhibitor of Ca^2+^ release from the ER (40). The increase of Ca^2+^ due to IT yeast treatment of mice bearing skin cancer is mainly due to Ca^2+^ released from the ER and Golgi of cancer cells, and not to the presence of intracellular Ca^2+^ from the yeast, since the amount of Ca^2+^ in the cytosol of yeast is 50–200 nM and therefore considered negligible (41–43). In addition, we measured the Ca2+ concentration in the yeast solution at concentration of 10^7^ cells/ml and found it to be negligible compared to the Ca2+ concentration in the tumor tissue.

Apoptosis (programmed cell death) is a physiologic form of cell death that plays an important role in normal development, tissue homeostasis, and pathological situations (44,45). There are two major pathways of apoptosis: 1) the mitochondrial (intrinsic) pathway, and 2) the death receptor (extrinsic) pathway (34, 46). A key feature of the intrinsic pathway of apoptosis is the disruption of mitochondrial membrane potential (MMP) and release of cytochrome-c, which activates the caspase cascade leading to apoptosis. Ca^2+^ triggers apoptotic signals directly via the mitochondrial pathway of apoptosis [34–36]. Ca^2+^ appears to facilitate disruption of MMP via activation of permeability transition pores (PTP) which ultimately results in the cell apoptosis (47,48). Our earlier study showed that phagocytosis of yeast by human breast MCF-7 cancer cells led to disruption of MMP [18]. In this study, the increase of Ca^2+^ post treatment with yeast was associated with an increase in the levels of both cytochrome-c and the expression of caspases 9, 8, and 3 in a dose-dependent manner, relative to mice bearing tumor alone.

Anti-apoptotic and pro-apoptotic effectors can modulate the mitochondrial pathway of apoptosis, and it is widely accepted that the predominance of the pro-apoptotic protein (Bax) over the anti-apoptotic protein (Bcl-2) promotes apoptosis (49, 50). Results of this study showed significant decrease in the expression of Bcl-2 and an increase in the Bax level. The effect was dose dependent relative to animals with tumor alone. These results coincide with the previous results that showed yeast-induced apoptosis in breast cancer, MDA-MB-231 cells, was associated with an increase in Bax and a substantial decrease in expression of Bcl-2 resulting in alteration in the Bax:Bcl-2 ratio (40), suggesting that yeast induces apoptosis of MDA-MB-231 cells *in vitro* by a mechanism involving intracellular Ca^2+^ and Bax:Bcl-2. Several studies showed that Bcl-2 and Bax can modulate Ca^2+^ mobilization from the ER to the cytosol and mitochondria during apoptosis through different mechanisms, including interaction with InsP(3) receptors (51, 52).

Fas-ligand (FasL), a member of the tumor necrosis superfamily, plays a central role in the induction of the apoptotic effect of CD8+ T cells and NK cells against cancer cells (53,54). FasL is strongly expressed in many types of cancer cells, and is considered a mechanism by which cancer cells can escape the attack of the immune system (55,56). The engagement of Fas/FasL interaction in the phenomenon of escape strategy has been reported in many types of malignancies, including hepatocellular carcinoma (57), endometrial cancer (58), leukemic cells (59, 60), and lung cancer (61). Similar observations were also described in melanoma and non-melanoma skin cancer (55, 56). Data of this study shows a significant increase in the level of FasL in tumor as compared with normal control skin (*P*≤0.001). However, mice bearing tumor that were treated with yeast showed significant decrease in the expression of FasL. This was associated with a significant increase in the expression of caspases 8 and 3 in a dose-dependent fashion as compared to mice bearing tumor alone. Caspase 8 is the initiator of the cascade reaction of cell apoptosis. It can transmit apoptotic signals and activate downstream effector caspase to induce cell apoptosis. This suggests that yeast can enhance apoptosis in skin cancer cells by downregulating FasL levels.

Taken together, yeast treatment resulted in a decrease in the expression of both FasL and Bcl2, and an increase in the Bax level relative to animals with tumor alone. In addition, yeast-treated mice showed an increase in the level of cytochrome-c as well as an increase in the expression of caspases 9, 8, and 3. This suggests that yeast treatment induces apoptosis in skin cancer cells by both the mitochondrial pathway and the death receptor pathway.

The yeast-induced apoptotic effect on tumor cells was associated with histopathological changes of the tumors. Yeast-treated mice, in a dose-dependent manner, showed significant histopathological tumor regression changes as compared with mice bearing tumor alone. Yeast-treated mice receiving high doses(10^9^cells/ml) showed a decrease in the thickness of the epidermal cell layers. The lining consisted of welldifferentiated keratinocytes and foci of glassy keratinocytes. Diminished keratin and marked regression of atypia with absence of dysplasia, mitosis, and polymorphism was also seen. These histopathological changes reflect the ability of yeast to induce an apoptotic effect on skin tumors.

It is of interest to note that *S. cerevisiae* can act as a chemosensitizer. Our recent study showed that Baker’s yeast in the presence of chemotherapy (paclitaxel) in vitro increased the sensitivity of metastatic murine 4T1 cells to chemotherapy (62). This exemplifies the additional capability of yeast to act as an anti-cancer agent.

The toxic effect triggered by administration of any foreign substance to animals or humans is a very important consideration. In our earlier studies, we examined the *in vitro* effects of Baker’s yeast against non-tumorgenic human fibrocystic mammary tissue cells, MCF-10A, and found that these cells did not exert phagocytic ability and yeast did not induce an apoptotic effect on that particular cell line (26). This suggests that the apoptotic effect of yeast is a selective phenomenon that is directed mainly towards phagocytic cells including cancer cells. Other studies on animals have revealed that animals can tolerate relatively high doses of yeast without any pathology detected up to at least 21 days post treatment, including rats (63), mice (64) and monkeys (65). The effect of yeast on humans has also been studied. For example, i.v. injections of yeast Glucans have been given to humans to boost the immune system of patients undergoing major surgery (66, 67) patients with *Paracoccidioidomycosis (PCM),* an endemic disease in most Latin American countries; and for elimination of P. *brasiliensis* (68). Results of these studies revealed no adverse side effects associated with yeast infusion (66, 69). In the current study, we have examined the potential toxic effect of yeast on mice bearing skin cancer, and the following observations were recorded: 1) animals treated with yeast IT survived the 16-week treatment period with normal feeding/drinking and life activity patterns, 2) histopathology of different organs derived from yeast-treated mice was similar to normal control mice, 3) biochemical analysis of yeast-treated mice, which included liver function, kidney function, and other parameters, were within the limits of normal healthy mice, and 4) changes in body weight showed that treatment with yeast resulted in counteracting the significant decrease in body weight due to cancer. Weight loss is common among people with cancer, and it has been also noticed in animal studies that adipose tissue wasting can occur as soon as the tumor is palpable (70).

In conclusion, Baker’s yeast (*S. cerevisiae*) is a safe, non-toxic agent that exerts anti-skin cancer activity in mice via intrinsic and extrinsic apoptotic pathways. This may suggest its possible use for the treatment of skin cancer in humans after investigations by clinical trials.

## Figures and Tables

**Table 1.** Effect of yeast treatment on serum aspartate aminotransferase (AST), serum alanine aminotransferase (ALT), blood urea, creatinine and alkaline phosphatase. Normal healthy mice were treated with yeast (100 μl) at different concentrations (10^7^, 10^8^, 10^9^ cells/l). One-way ANOVA with Dunnett post hoc tests were used to control for alpha-error inflation for multiple comparisons.

Parameter	Normal Control mice	Tumor bearing mice			
		Untreated	Yeast 10^9	Yeast 10^8	Yeast 10^7
AST U/l	117.4±13.48	176.3±4.94 ϕ	99.6±3.33 †ŧ	109±9.11 †	114±9.27**
ALT U/l	38.6±5.87	95.5±5.80 ϕ	32.8±5.50 †Ω ŧ	54±8.15 *	54±8.09*
ALP U/l	66.2±0.22	93.7±2.08 ‡	45.2±6.64 †	54.4±6.77**	57.4±9.54**
Urea mg/dl	17.6±4.82	42.5±3.81 £	14.6±3.09 †	18.2±3.63**	20.6±4.26**
Creatinine mg/dl	.46±.13	1.026±.03 ϕ	.5±.14 **	.52±.13**	.58±.13**

£ p≤0.0001-significant to normal control mice.

ϕ p≤0.001 - significant to normal control mice.

‡ p≤0.05 - significant to normal control mice.

† p≤0.0001-significant to untreated tumor bearing mice.

** p≤0.001 - significant to untreated tumor bearing mice.

* p≤0.05 - significant to untreated tumor bearing mice.

Ω p≤0.01- significant to yeast-treated tumor bearing mice at doses 10^7^ and 10^8^ cells/l.

ŧ p≤0.05 - significant to yeast-treated tumor bearing mice at dose 10^7^ cells/l.

**Table 2.** Effect of yeast treatment on lipid peroxide (nmol/ml). One-way ANOVA with Dunnett post hoc tests were used to analyse data. Significance was defined by a p-value ≤ 0.05.

Groups	Mean±S.E	Compared to Normal Control mice	Compared to Tumor bearing mice
Normal Control	43.59±1.95		*p*≤0.001
Untreated Tumor bearing mice	656.38±62.99	*p*≤0.001	
Yeast 10^7^	99.88±3.06	*p*≤0.001	*p*≤0.001
Yeast 10^8^	63.12±3.97	*p*≤0.01	*p*≤0.001
Yeast 10^9^	47.4±3.09	*p*≤0.05	*p*≤0.001

## Cogent Medicine

### Public interest statement:

Baker’s yeast is an essential component for the production of fermented foods like bread and beer. Our earlier studies showed that heat-killed baker’s yeast exerts anticancer activity against different types of cancer. This is based on the observation that cancer cells can engulf yeast, which ultmately shuts down and kills the cancer cell from within. This study aims to assess baker’s yeast as a novel natural product that can cause the death of skin cancer cells in animals. Several parameters were examined and show skin tumors stopped growing and regressed in mice treated with yeast compared to those untreated. Though several treatments for skin cancer exist, these are known to have severe side effects. We show the anticancer effects of yeast as a safe, non-toxic agent, which may suggest its possible use as treatment against skin cancer in humans.

## Cogent Medicine

### Author statement:

Dr. Ghoneum is an internationally recognized immunologist, who is an expert in the area of cancer immune therapy. He has published over 100 articles and 130 abstracts, as well as 11 books in Japanese and in English related to cancer. He holds 2 patents for 2 biological response modifiers (BRMs) for the treatment of cancer. Dr. Ghoneum’s contribution to immunotherapy has been to introduce several new non-toxic BRMs that activate different arms of the immune system and induce the death of cancer cells. Another approach he discovered is the use of heat-killed Baker’s yeast as a novel therapy for the treatment of cancer. Most recently, Dr. Ghoneum showed that Baker’s yeast is an effective chemosensitizer and can enhance the killing capacity of the chemotherapeutic agent paclitaxel against breast cancer cells. Baker’s yeast may represent a novel, non-toxic adjuvant for chemotherapy treatment.

## References

[1] https://www.cancer.org/research/cancer-facts-statistics/all-cancer-facts-figures/cancerfacts-figures-2017.html. Accessed 12 March 2017.

[2] http://www.cancer.org.au/about-cancer/types-of-cancer/skin-cancer.html. Accessed 30 April 2016.

[3] Sosman JA, Puzanov I. Molecular targets in melanoma from angiogenesis to apoptosis. Clin Cancer Res 2006; 12(7 Pt 2): 2376s–2383s. 16609062 10.1158/1078-0432.CCR-05-2558

[4] Gartel AL. Mechanisms of apoptosis induced by anticancer compounds in melanoma cells. Curr Top Med Chem 2012;12: 50–52. 22280162 10.2174/156802612798919196

[5] Mocellin S, Lens MB, Pasquali S, Pilati P, Chiarion Sileni V. Interferon alpha for the adjuvant treatment of cutaneous melanoma. Cochrane Database Syst Rev 2013; 6: CD008955. 10.1002/14651858.CD008955.pub2 PMC10773707 23775773

[6] La-Beck NM, Jean GW, Huynh C, Alzghari SK, Lowe DB. Immune Checkpoint Inhibitors: New Insights and Current Place in Cancer Therapy. Pharmacotherapy 2015; 35: 963–976. 26497482 10.1002/phar.1643

[7] Bourke JM, O’Sullivan M, Khattak MA. Management of adverse events related to new cancer immunotherapy (immune checkpoint inhibitors). Med J Aust 2016; 205: 418–424. 27809739 10.5694/mja16.00586

[8] D Aniello C, Perri F, Scarpati GD, Pepa CD, Pisconti S, Montesarchio V, Melanoma Adjuvant Treatment: current insight and clinical features. Curr Cancer Drug Targets 2017; doi: 10.2174/1568009617666170208163714 [Epub ahead of print] 28183255

[9] Bertrand A, Kostine M, Barnetche T, Truchetet ME, Schaeverbeke T. Immune related adverse events associated with anti-CTLA-4 antibodies: systematic review and metaanalysis. BMC Med 2015;13: 215–225. 26337719 10.1186/s12916-015-0455-8 PMC4559965

[10] Horvat TZ, Adel NG, Dang TO, Momtaz P, Postow MA, Callahan MK, Immunerelated adverse events, need for systemic immunosuppression, and effects on survival and time to treatment failure in patients with melanoma treated with Ipilimumab at Memorial Sloan Kettering Cancer Center. J Clin Oncol 2015; 33: 3193–3198. 26282644 10.1200/JCO.2015.60.8448 PMC5087335

[11] Lipton A, Harvey HA, Lawrence B, Gottlieb R, Kukrika M, Dixon R, Corynebacterium parvum versus BCG adjuvant immunotherapy in human malignant melanoma. Cancer 1983;51:57–60. 6821809 10.1002/1097-0142(19830101)51:1<57::aid-cncr2820510114>3.0.co;2-v

[12] Lipton A, Harvey HA, Balch CM, Antle CE, Heckard R, Bartolucci AA. Corynebacterium parvum versus bacille Calmette-Guérin adjuvant immunotherapy of stage II malignant melanoma. J Clin Oncol 1991; 9:1151–1156. 2045856 10.1200/JCO.1991.9.7.1151

[13] Fahey JL, Brosman S, Ossorio RC, O’Toole C, Zighelboim J. Immunotherapy and human tumor immunology. Ann Intern Med 1976; 84:454–465. 1259292 10.7326/0003-4819-84-4-454

[14] Hilal EY, Pinsky CM, Hirshaut Y, Wanebo HJ, Hansen JA, Braun DW Jr, Surgical adjuvant therapy of malignant melanoma with corynebacterium parvum. Cancer 1981; 48:245–251. 7016302 10.1002/1097-0142(19810715)48:2<245::aid-cncr2820480206>3.0.co;2-h

[15] Fritze D, Massner B, Becher R, Kaufmann M, Illiger HJ, Hartlapp J, Combination chemotherapy (VAC/FMC) with immunostimulation in metastatic breast cancer: a randomized study comparing different times and routes of administration of Corynebacterium parvum. Klin Wochenschr 1984; 62:162–167. 6368960 10.1007/BF01731638

[16] Hirshaut Y, Pinsky CM, Wanebo HJ, Braun DW Jr. Corynebacterium parvum toxicity in patients with limited and advanced malignancy. Eur J Cancer Clin Oncol 1984; 20:583591. 10.1016/0277-5379(84)90002-6 6539697

[17] Vázquez-Lavista LG, Flores-Balcázar CH, Llorente L. The bacillus Calmette-Guérin as immunomodulator in bladder cancer. Rev Invest Clin 2007; 59:146–152. 17633803

[18] Ghoneum M, Gollapudi S. Induction of apoptosis in human breast cancer cells by Saccharomyces cerevisiae, the baker’s yeast, in vitro. Anticancer Res 2004; 24: 1455–1463. 15274310

[19] Ghoneum M, Wang L, Agrawal S, Gollapudi S. Yeast therapy for the treatment of breast cancer: a nude mice model study. In Vivo 2007; 21: 251–258. 17436573

[20] Abel EL, Angel JM, Kiguchi K, DiGiovanni J. Multi-stage chemical carcinogenesis in mouse skin: fundamentals and applications. Nat Protoc. 2009; 4(9):1350–62 19713956 10.1038/nprot.2009.120 PMC3213400

[21] Wang Y, Auyeung KK, Zhang X, Ko JK1 Astragalus saponins modulates colon cancer development by regulating calpain-mediated glucose-regulated protein expression. BMC Complement Altern Med. 2014; 14:401. 25319833 10.1186/1472-6882-14-401 PMC4210535

[22] Satoh K Serum lipid peroxide in cerebrovascular disorders determined by a new colorimetric method. Clinica Chimica Acta 1978; 90: 37–43. 10.1016/0009-8981(78)90081-5 719890

[23] Bartles H, Bohmer M, Heirli C. Serum creatinine determination without protein precipitation. Clinica Chemica Acta 1972; 37: 193–197. 10.1016/0009-8981(72)90432-9 5022083

[24] Belfield A, Goldberg DM. Revised assay for serum phenyl phosphatase activity using 4amino-antipyrine. Enzyme 1971; 12: 561–573. 5169852 10.1159/000459586

[25] Fawcett JK, Scott JE. A rapid and precise method for determination of urea. J Clinical Pathol 1960; 13: 156–159. 13821779 10.1136/jcp.13.2.156 PMC480024

[26] Ghoneum M, Gollapudi S. Modified arabinoxylan rice bran (MGN-3/Biobran) enhances yeast-induced apoptosis in human breast cancer cells in vitro. Anticancer Res 2005;25: 859870. 15868920

[27] Ghoneum M, Gollapudi S. Synergistic role of arabinoxylan rice bran (MGN-3/Biobran) in S. cerevisiae-induced apoptosis of monolayer breast cancer MCF-7 cells. Anticancer Res 2005; 25: 3–12. 16309215

[28] Ghoneum M, Hamilton J, Brown J, Gollapudi S. Human squamous cell carcinoma of the tongue and colon undergoes apoptosis upon phagocytosis of Saccharomyces cerevisiae, the baker’s yeast, in vitro. Anticancer Res 2005; 25: 981–989. 15868937

[29] Ghoneum M, Brown J, Gollapudi S. Yeast therapy for the treatment of cancer and its enhancement by MGN-3/Biobran, an arabinoxylan rice bran Cellular Signaling and Apoptosis Research. Demasi AR(ed.) 2007, Nova Science Publishers, Inc., Hauppauge, New York, p 185–200.

[30] Ghoneum M, Badr El-Din NK, Noaman E, Tolentino L. Saccharomyces cerevisiae, the Baker’s yeast, suppresses the growth of Ehrlich carcinoma-bearing mice. Cancer Immunol Immunother 2008; 57: 581–592. 17891396 10.1007/s00262-007-0398-9 PMC11030098

[31] Neumann C, Macher E, Sorg C. Interferon production by Corynebacterium parvum and BCG-activated murine spleen macrophages. Immunobiology 1980; 157(1):12–23. 6162779 10.1016/S0171-2985(80)80057-X

[32] Mori H, Mihara M, Uesugi Y, Nagai H, Koda A. Mechanism for macrophage activation against Corynebacterium parvum--participation of T cells and its lymphokines. Microbiol Immunol 1994; 38:983–938. 7723692 10.1111/j.1348-0421.1994.tb02156.x

[33] Simons MP, O’Donnell MA, Griffith TS. Role of neutrophils in BCG immunotherapy for bladder cancer. Urol Oncol 2008; 26:341–345. 18593617 10.1016/j.urolonc.2007.11.031 PMC2493065

[34] Green DR, Reed JC. Mitochondria and apoptosis. Science 281: 1309–1312, 1998. 9721092 10.1126/science.281.5381.1309

[35] Smaili SS, Hsu YT, Youle RJ, Russell JT. Mitochondria in Ca^2+^ signaling and apoptosis. J Bioenerg Biomembr 2000; 32: 35–46. 11768760 10.1023/a:1005508311495

[36] Bernardi P, Rasola A. Calcium and cell death: the mitochondrial connection. Subcell Biochem 2007; 45:481–506. 18193649 10.1007/978-1-4020-6191-2_18

[37] Di Virgilio F, Meyer BC, Greenberg S, Silverstein SC. Fc receptor-mediated phagocytosis occurs in macrophages at exceedingly low cytosolic Ca^2+^ levels. J Cell Biol 1988; 106: 657–666. 3346321 10.1083/jcb.106.3.657 PMC2115077

[38] Greenberg S, El Khoury J, Di Virgilio F, Kaplan EM, Silverstein SC. Ca^2+^-independent Factin assembly and disassembly during Fc receptor-mediated phagocytosis in mouse macrophages. J Cell Biol 1991; 113: 757–767. 2026648 10.1083/jcb.113.4.757 PMC2288985

[39] Myers JT, Swanson JA. Calcium spikes in activated macrophages during Fc-receptormediated phagocytosis. J Leukoc Biol 2002; 72: 677–684. 12377936

[40] Ghoneum M, Matsuura M, Braga M, Gollapudi S. S. cerevisiae induces apoptosis in human metastatic breast cancer cells by altering intracellular Ca^2+^ and the ratio of Bax and Bcl-2. Int J Oncol 2008; 33: 533–539. 18695883

[41] Dunn T, Gable K, Beeler T. Regulation of Cellular Ca^2+^ by Yeast Vacuoles. J. Biol. Chem 1994; 269: 7273–7278. 8125940

[42] Miseta A, Kellermayer R, Aiello DP, Fu L, Bedwell DM. The vacuolar Ca^2+^/H+ exchanger Vcx1p/Hum1p tightly controls cytosolic Ca^2+^ levels in S. cerevisiae. FEBS Letters 1999; 451: 132–136. 10371152 10.1016/s0014-5793(99)00519-0

[43] Aiello DP, Fu L, Miseta A, Bedwell DM. Intracellular Glucose 1-Phosphate and Glucose 6Phosphate Levels Modulate Ca^2+^ Homeostasis in Saccharomyces cerevisiae. J Biol Chem 2002; 277: 45751–45758. 12351653 10.1074/jbc.M208748200

[44] Kerr JF, Wyllie AH, Currie AR. Apoptosis: a biological phenomena with wide-ranging implications in tissue kinetics. Brit J Cancer 1972; 26: 239–257. 4561027 10.1038/bjc.1972.33 PMC2008650

[45] Arends MJ, Wyllie AH. Apoptosis: mechanisms and roles in pathology. Rev Exp Pathol Int 1991; 32: 223–254. 10.1016/b978-0-12-364932-4.50010-1 1677933

[46] Gupta S Molecular steps of Tumor necrosis factor receptor-mediated apoptosis. Curr Mol Med 2001; 1: 299–306. 10.2174/1566524013363780 11899080

[47] Bernardi P Mitochondrial transport of cations: channels, exchangers, and permeability transition. Physiol Rev 1999; 79: 1127–1155. 10508231 10.1152/physrev.1999.79.4.1127

[48] Kroemer G, Reed JC. Mitochondrial control of cell death. Nat Med 2000; 6: 513–519. 10802706 10.1038/74994

[49] Salomons GS, Brady HJ, Verwijs-Janssen M, Van Den Berg JD, Hart AA, Van Den Berg H, The Bax:Bcl-2 ratio modulates the response to dexamethasone in leukaemic cells and is highly variable in childhood acute leukaemia. Int J Cancer 1997; 71: 959–965. 9185697 10.1002/(sici)1097-0215(19970611)71:6<959::aid-ijc9>3.0.co;2-x

[50] Wolter KG, Hsu YT, Smith CL, Nechushtan A, Xi XG, Youle RJ. Movement of Bax from the cytosol to mitochondria during apoptosis. J Cell Biol 1997; 139: 1281–1292. 9382873 10.1083/jcb.139.5.1281 PMC2140220

[51] Lao Y, Chang DC. Study of the functional role of Bcl-2 family proteins in regulating Ca^2+^ signals in apoptotic cells. Biochem Soc Trans 2007; 35(Pt 5): 1038–1039. 17956272 10.1042/BST0351038

[52] Lewis A, Hayashi T, Su TP, Betenbaugh MJ. Bcl-2 family in inter-organelle modulation of calcium signaling; roles in bioenergetics and cell survival. J Bioenerg Biomembr 2014; 46: 1–15. 24078116 10.1007/s10863-013-9527-7 PMC4529064

[53] Kojima Y, Kawasaki-Koyanagi A, Sueyoshi N, Kanai A, Yagita H, Okumura K. Localization of Fas ligand in cytoplasmic granules of CD8+ cytotoxic T lymphocytes and natural killer cells: participation of Fas ligand in granule exocytosis model of cytotoxicity. Biochem Biophys Res Commun 2002; 296: 328–336. 12163021 10.1016/s0006-291x(02)00841-0

[54] Mehmut M, Takeda K, Abe M, Ogata H, Hirose S, Okumura K, Fas ligand and TNF-related apoptosis-inducing ligand induction on infiltrating lymphocytes in bladder carcinoma by bacillus Calmette-Guérin treatment. Urol Int 2005; 75: 80–87. 16037714 10.1159/000085934

[55] Massari LP, Kastelan M, Gruber F. Epidermal malignant tumors: pathogenesis, influence of UV light and apoptosis. Coll Antropol 31 Suppl 2007; 1: 83–85. 17469758

[56] Erb P, Ji J, Kump E, Mielgo A, Wernli M. Apoptosis and pathogenesis of melanoma and nonmelanoma skin cancer. Adv Exp Med Biol 2008; 624: 283–295. 18348464 10.1007/978-0-387-77574-6_22

[57] Fabregat I: Dysregulation of apoptosis in hepatocellular carcinoma cells. World J Gastroenterol 15: 513–520, 2009. 19195051 10.3748/wjg.15.513 PMC2653340

[58] Chaudhry P, Asselin E: Resistance to chemotherapy and hormone therapy in endometrial cancer. Endocr Relat Cancer 16: 363–380, 2009. 19190080 10.1677/ERC-08-0266

[59] Komada Y, Sakurai M: Fas receptor (CD95)-mediated apoptosis in leukemic cells. Leuk Lymphoma 25: 9–21, 1997. 9130610 10.3109/10428199709042492

[60] Wang L, Zhao S, Wang HX, Zou P: Inhibition of NF-kappa B can enhance Fas-mediated apoptosis in leukemia cell line HL-60. Front Med China 4: 323–328, 2010. 21191839 10.1007/s11684-010-0026-5

[61] Huang G, Nishimoto K, Yang Y, Kleinerman ES: Participation of the Fas/FasL signaling pathway and the lung microenvironment in the development of osteosarcoma lung metastases. Adv Exp Med Biol 804: 203–217, 2014. 24924176 10.1007/978-3-319-04843-7_11

[62] Badr El-Din NK, Mahmoud AZ, Hassan TA, Ghoneum M: Baker’s yeast sensitizes metastatic breast cancer cells to paclitaxel in vitro. Integr Cancer Ther 1534735417740630, 2017. 10.1177/1534735417740630 PMC6041900 29161917

[63] Fahrig R Development of host-mediated mutagenicity tests-yeast systems. II. Recovery of yeast cells out of testes, liver, lung, and peritoneum of rats. Mutat Res 1975;31:381–94. 768756 10.1016/0165-1161(75)90048-5

[64] Frezza D, Zeiger E, Gupta BN. The intrasanguineous host-mediated assay procedure distribution and retention of yeast in the mouse. Mutat Res 1979; 64:295–305. 390379 10.1016/0165-1161(79)90122-5

[65] Maejima K, Shimoda K, Morita C, Fujiwara T, Kitamura T. Colonization and pathogenicity of Saccharomyces cerevisiae, MC16, in mice and cynomolgus monkeys after oral and intravenous administration. Jpn J Med Sci Biol 1980;33:271–6. 7029035 10.7883/yoken1952.33.271

[66] Babineau TJ, Marcello P, Swails W, Kenler A, Bistrian B, Forse RA. Randomized phase I/II trial of a macrophage-specific immunomodulator (PGG-glucan) in high-risk surgical patients. Ann Surg 1994; 220: 601–9. 7979607 10.1097/00000658-199411000-00002 PMC1234447

[67] Babineau TJ, Hackford A, Kenler A, Bistrian B, Forse RA, Fairchild PG, A phase II multicenter, double-blind, randomized, placebo-controlled study of three dosages of an immunomodulator (PGG-glucan) in high-risk surgical patients. Arch Surg 1994; 129: 1204–10. 7979954 10.1001/archsurg.1994.01420350102014

[68] Wanke B, Londero AT. Epidemiology and paracoccidioidomycosis infection, in Paracoccidioidomycosis, Franco M, Lacaz CS, Restrepo-Moreno A, and Del Negro G, Editors. 1994, CRC Press: Boca Raton p. 109–120.

[69] Meira DA, Pereira PC, Marcondes-Machado J, Mendes RP, Barraviera B, Pellegrino Junior J, The use of glucan as immunostimulant in the treatment of paracoccidioidomycosis. Am J Trop Med Hyg 1996; 55: 496–503. 8940980 10.4269/ajtmh.1996.55.496

[70] Costa G, Holland JF. Effects of Krebs-2 carcinoma on the lipid metabolism of male Swiss mice. Cancer Res 1962; 22: 1081–1083. 14023263

